# Supplementary material for: Association between COVID-19 risk-mitigation behaviors and specific mental disorders in youth
Source: Child Adolesc Psychiatry Ment Health. 2023 Jan 24;17:14. doi: 10.1186/s13034-023-00561-7 (PMC9872749; doi:10.1186/s13034-023-00561-7)
Supplement: Supplementary file 1 — Additional file 1: Table S1. Sample Characteristics by Study Completion Status. [file 13034_2023_561_MOESM1_ESM.docx]

**Additional file 1: Sample Characteristics by Study Completion Status**

|  | **Sample Characteristics** | | | | |
| --- | --- | --- | --- | --- | --- |
|  |  | | | | |
|  | Invited to Participate | Collected and Matched to HBN | Completed  Survey | Completed HBN Diagnosis | Total Analytic  Sample |
| **Sex** |  |  |  |  |  |
| Male | 1131 (64%) | 645 (62%) | 590 (62%) | 550 (62%) | 514 (62%) |
| Female | 649 (36%) | 393 (38%) | 364 (38%) | 332 (38%) | 314 (38%) |
| **Age (years)** |  |  |  |  |  |
| 5-6 | 118 (7%) | 26 (3%) | 25 (3%) | 21 (2%) | 20 (2%) |
| 7-9 | 565 (32%) | 277 (27%) | 252 (26%) | 229 (26%) | 217 (26%) |
| 10-12 | 552 (31%) | 364 (35%) | 340 (36%) | 321 (36%) | 302 (36%) |
| 13-15 | 302 (17%) | 221 (21%) | 200 (21%) | 185 (21%) | 178 (21%) |
| 16+ | 243 (14%) | 150 (14%) | 137 (14%) | 126 (14%) | 111 (13%) |
| **Family Structure** |  |  |  |  |  |
| Single caregiver | 156 (9%) | 95 (9%) | 84 (9%) | 82 (9%) | 70 (8%) |
| **SES**^1^ |  |  |  |  |  |
| Low | 127 (7%) | 79 (8%) | 71 (7%) | 63 (7%) | 58 (7%) |
| Middle | 328 (18%) | 193 (19%) | 178 (19%) | 165 (19%) | 153 (18%) |
| High | 1255 (71%) | 731 (70%) | 677 (71%) | 649 (74%) | 617 (75%) |
| **Race** |  |  |  |  |  |
| Caucasian | 945 (53%) | 525 (51%) | 491 (51%) | 471 (53%) | 444 (54%) |
| African American | 197 (11%) | 127 (12%) | 115 (12%) | 108 (12%) | 99 (12%) |
| Hispanic | 150 (8%) | 94 (9%) | 82 (9%) | 76 (9%) | 71 (9%) |
| Asian | 50 (3%) | 31 (3%) | 29 (3%) | 28 (3%) | 27 (3%) |
| Other | 319 (18%) | 200 (19%) | 181 (19%) | 166 (19%) | 158 (19%) |
| Unknown | 119 (7%) | 61 (6%) | 56 (6%) | 33 (4%) | 29 (4%) |
| **Site**^2^ |  |  |  |  |  |
| Staten Island^2^ | 653 (37%) | 366 (35%) | 331 (35%) | 308 (35%) | 288 (35%) |
| Midtown | 467 (26%) | 257 (25%) | 235 (25%) | 226 (26%) | 210 (25%) |
| Harlem | 650 (37%) | 406 (39%) | 379 (40%) | 339 (38%) | 322 (39%) |

Note: ^1^ Barratt total score was divided into tertiles: Low (3-24), medium (25-45), high (46-66), and missing Barratt Score is not shown in table (N=70, %=4). ^2^ Mobile Research Vehicle (MRV) site not shown in table (N=10, %=1). ^3^ Combined Staten Island site and Staten Island Richmond University Medical Center site.
